# Association between the IL-10-1082G/A, IL-10-819T/C and IL-10-592A/C polymorphisms and Brucellosis susceptibility: a meta-analysis

**DOI:** 10.1017/S0950268819002036

**Published:** 2019-12-11

**Authors:** Xiaochun Jin, Shuzhou Yin, Youtao Zhang

**Affiliations:** 1 Department of Anesthesiology, Suzhou Kowloon Hospital, Shanghai Jiaotong University School of Medicine, Suzhou, 215028, People's Republic of China; 2 Department of Clinical Laboratory, First Affiliated Hospital of Soochow University, Suzhou, 215006, People's Republic of China

**Keywords:** Brucellosis, interleukin-10, meta-analysis, polymorphism

## Abstract

Brucellosis is a widespread zoonosis caused by small bacteria of the genus *Brucella*. The promoter polymorphisms of IL-10 (-1082 loci, -819 loci and -590 loci) are closely related to the production of IL-10, leading to the alteration of development and pathogenesis of Brucellosis. However, the previous results were controversial. In the present study, we conduct the meta-analysis to get a more precise result of IL-10 polymorphisms with Brucellosis risk. The quality of the studies was assessed according to a predefined scale. The odds ratio (OR) and 95% confidence interval (CI) were counted to evaluate the association strength. No significant association was found between position -1082 loci or -590 loci polymorphism and Brucellosis risk. The significant association was found in Asian population of position -819 (T *vs.* C: OR 0.60, 95% CI 0.44–0.82, *P* = 0.001), homozygote comparison (TT *vs.* CC: OR 0.24, 95% CI 0.09–0.62, *P* = 0.003) and recessive genetic model (TT *vs.* TC/CC: OR 0.22, 95% CI 0.05–0.91, *P* = 0.036). The present meta-analysis demonstrates that IL-10-819 loci polymorphism is not associated with Brucellosis risk of Caucasian population but may contribute a decreased risk to Asian population. And neither IL-10-1082 loci nor -592 loci polymorphism is associated with Brucellosis risk.

## Introduction

As a major and widespread zoonosis, Brucellosis results from the genus *Brucella* bacteria. Although this morbidity is not high in plentiful developed countries, it is still a severe health issue and has been endemic in various developing countries and regions including Asia, Africa, the Mediterranean and the Middle East [1, 2]. This disease has variety of clinical manifestations such as fatigue, fever, arthralgia and sweating. Its diagnosis is not easy when the clinical presentation is not typical.

So far, the mechanism of host resistance to Brucellosis has not been well clarified. However, cellular immunity is deemed to act a crucial function in immunity to the invasion of Brucellosis [3]. Th2 cytokines are able to restrain a macrophage effect of IFN-*γ* and hold back the reaction of cellular immunity. Interleukin-10 (IL-10) is a crucial component of Th2 cytokine. What is more, it can lead to the reduction of IFN-*γ* production. Additionally, the generation of many cytokines is controlled by inheritance factors and cytokine polymorphisms are possibly crucial which may be genetic predictors for disease susceptibility or clinical significance [4]. This feature is quite obvious in IL-10 gene polymorphism. IL-10 polymorphism possesses positions including -1082(G/A) locus, -819 (T/C) locus and -592 (A/C) locus, which are three important functional locus.

Considerable reports suggested that the promoter polymorphisms of IL-10 are closely related to the output of IL-10 and development and pathogenesis of multiple diseases, including Brucellosis. However, the results were controversial. At present, we conduct the meta-analysis to obtain more accurate results of IL-10 polymorphisms with Brucellosis susceptibility.

## Materials and methods

### Search strategy

Our present research was executed on the basis of the predefined protocol [5]. The Embase database, PubMed database and Chinese Biomedical Literature Database were searched applying these phrases: (‘IL-10’ or ‘Interleukin-10’) together with ‘Brucellosis’ updated on April 2019 for whole literatures of the relationship. There were no restrictions on original language, publication year, sample size, genotyping methods or source of control. All of the eligible studies were searched, reviewed and retrieved. The reference of each included study was also carefully reviewed for searching new corresponding literatures.

### Inclusion and exclusion criteria

Three issues of inclusion standards: (a) it is a case-control study or short communication; (b) it is a comparison of IL-10 polymorphism with Brucellosis susceptibility and (c) these literatures should provide sample size, genotypes frequency or other messages that can speculate the results. Accordingly, literatures were not approved if these standards below existed: (a) literatures which included repetitive data and (b) it doesn't provide adequate data to judge the relationship of IL-10 polymorphisms with Brucellosis susceptibility.

### Data extraction

All information was independently gathered by the authors (Shuzhou Yin and Xiaochun Jin) and the results were judged by the final referee (Youtao Zhang). The author name, nation, ethnics, genotyping method, genotyping frequency and source of control should be extracted as basic information. Ethnic groups should be categorised as Asian, Caucasian, or other populations. In order to guarantee the veracity of extracted data, two researchers (Shuzhou Yin and Xiaochun Jin) checked the existing data and information and reached an agreement. If there are different opinions, they would recheck the above information and discuss in order to reach consensus. If the controversial results still existed, the corresponding author (Youtao Zhang) will be invited to make final decisions.

### Methodological quality assessment

Methodological quality assessment was evaluated according to the opinions of authors (Shuzhou Yin and Xiaochun Jin) based on predefined assessment standard (Table 1) according to the terms of Jiang *et al*. [6]. The grades ranged from 0 (lowest) to 18 (highest) according to different evaluation extents including credibility of controls, matching degree, diagnosis criteria of Brucellosis, HWE conformity and sample size. All evaluation extents of methodological quality assessment were implemented by traditional epidemiological issues and characteristic of Brucellosis. Literatures whose grades <12 were regarded as studies named ‘low-quality’. Nevertheless, the literatures with grades ≥12 was regarded as studies named ‘high-quality’.

**Table 1.** The predefined assessment criteria of eligible studies

Evaluation criterion	Score
Credibility of controls	
Population-based from the same geographical area	3
Blood or organ donors or volunteers	2
Hospital-based with no history of Brucellosis	1
Not mentioned in literatures	0
Matching criteria	
Age, sex and ethnicity	3
Only matching with ethnicity	1.5
Not described	0
Diagnosis of Brucellosis	
Clinical discoveries with high titres of antibodies	3
Established by history or physical examination	1.5
Not mentioned in literatures	0
Genotyping examination	
Blinded condition of genotyping procedure	3
Not mentioned or unblinded	0
Hardy–Weinberg equilibrium	
Hardy–Weinberg equilibrium in controls	3
Hardy–Weinberg disequilibrium in controls	0
Total sample size	
>500	3
>200 and ⩽500	2
≥100 and ⩽200	1
<100	0

### Statistical analysis

Odds ratio (OR) and 95% confidence interval (CI) were counted to make an assessment of the association power in four different models, which comprised of allele comparison model, homozygote comparison model, recessive model and dominant model [7]. The *χ*^2^ test which is based on *Q*-statistic was used and *I*^2^ statistics was also put into use. In the event of evident heterogeneity, the random-effect model was going to be put into use [8]. If not, the fixed-effect model was going to be put into use [9]. Sensitivity analysis was going to be employed. Funnel plots and Egger's test were going to be employed for detecting possible publication bias [10]. The Stata software took responsibility for all statistics.

## Results

### Eligible studies

**Fig. 1.**
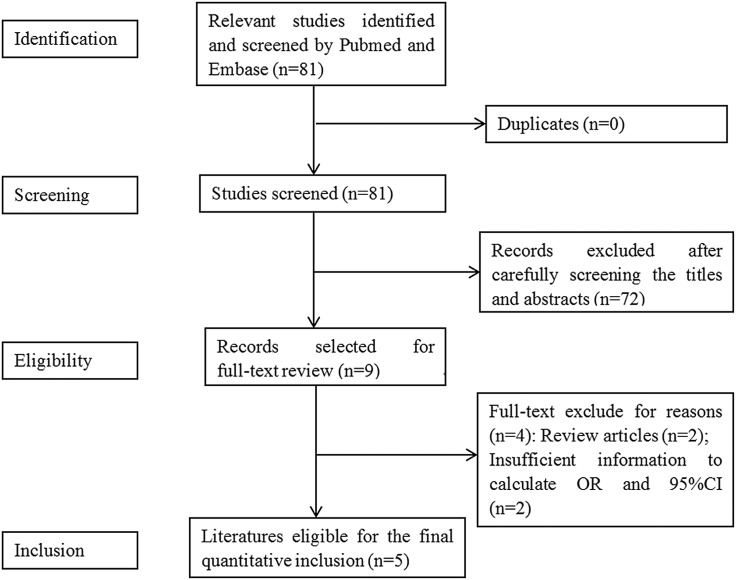
Flow diagram for identification of eligible studies for this meta-analysis.

Figure 1 shows our detailed search procedure. On the basis of the previous search method, five literatures met our requirements [11–15]. It should be noted that the genotyping data of two literatures were obtained by sending emails to authors. The main features of all studies which met our requirements were displayed in Table 2.

**Table 2.** Basic information of eligible studies

Literature	Ethnics (country)	Genotyping methods	Source of control	Sample size (case/control)	Studied polymorphisms	*P* value of HWE	Quality score
Bravo (2003)	Caucasian (Spain)	PCR-SSP	PB	83/101	IL-10(-1082, -819, -592)	0.435	14
Budak (2007)	Caucasian (Turkey)	PCR-SSP	PB	40/50	IL-10(-1082, -819, -592)	0.060	12
Rasouli (2008)	Asian (Iran)	PCR-RFLP	PB	190/81	IL-10(-1082, -819, -592)	0.989	13
Karaoglan (2009)	Caucasian (Turkey)	PCR-SSP	PB	85/85	IL-10(-1082, -819)	0.246	13
Kazemi (2016)	Asian (Iran)	PCR-RFLP	PB	60/60	IL-10(-1082, -819, -592)	0.320	13

PB, population-based; HWE, Hardy–Weinberg equilibrium in control population; PCR-SSP, polymerase chain reaction-sequence-specific primer.

PCR–RFLP, polymerase chain reaction-restriction fragment length polymorphism.

### Quantitative synthesis of data

**Fig. 2.**
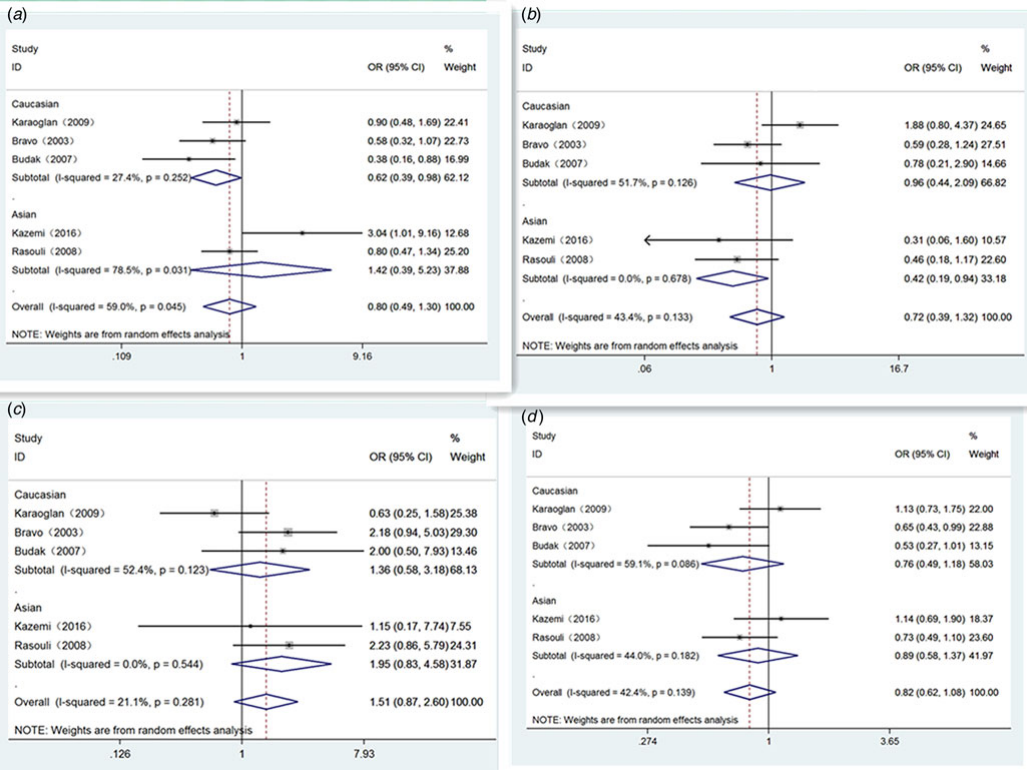
Forest plot of IL-10-819 loci polymorphism on Brucellosis risk in different genetic models. A: recessive model (TT *vs.* CC/TC); B: allele model (T *vs.* C); C: dominant model (TT/TC *vs.* CC) and D: homozygote model (TT *vs.* CC).

**Fig. 3.**
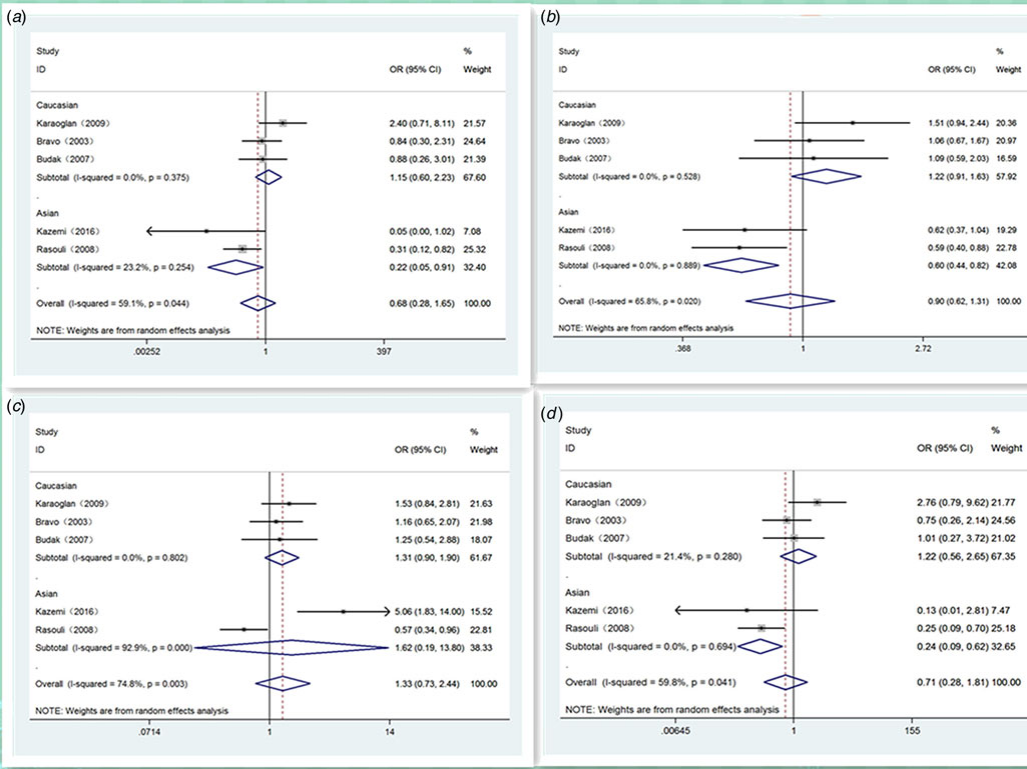
Forest plot of IL-10-1082 loci polymorphism on Brucellosis risk in different genetic models. A: recessive model (AA *vs.* GG/GA); B: dominant model (AA/GA *vs.* GG); C: homozygote model (AA *vs.* GG) and D: allele model (A *vs.* G).

**Fig. 4.**
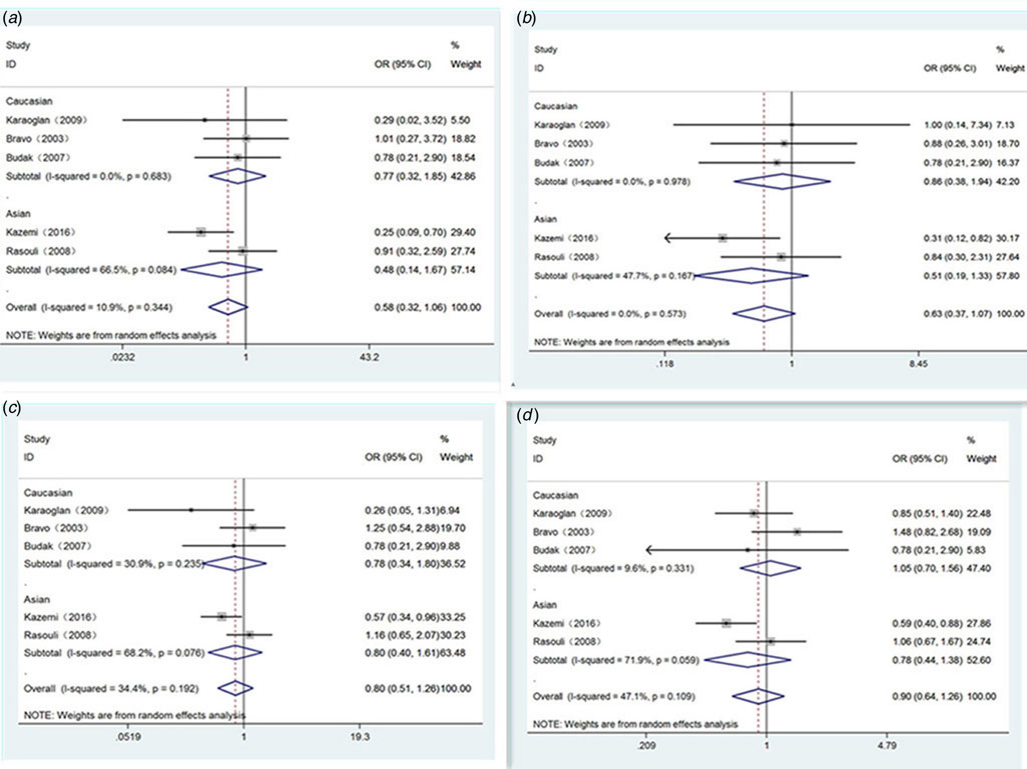
Forest plot of IL-10-592 loci polymorphism on Brucellosis risk in different genetic models. A: recessive model (AA *vs.* CC/AC); B: dominant model (AA/AC *vs.* CC); C: homozygote model (AA *vs.* CC) and D: allele model (A *vs.* C).

Table 3 shows our detailed results. Generally, significant relevance of IL-10 polymorphisms with Brucellosis sensibility was only found in Asian population of position -819 (T *vs.* C: OR 0.60, 95% CI 0.44–0.82, *P* = 0.001) (Fig. 2b), homozygote comparison genetic model (TT *vs.* CC: OR 0.24, 95% CI 0.09–0.62, *P* = 0.003) (Fig. 2d) and recessive genetic model (TT *vs.* TC/CC: OR 0.22, 95% CI 0.05–0.91, *P* = 0.036) (Fig. 2a). For position -1082 and position -592, there were no significant relationship in any population (Figs 3 and 4). Haplotype analysis displayed a very striking association between GCC haplotype and Brucellosis susceptibility (GCC *vs.* ACC: OR 1.62, 95% CI 1.07–2.46, *P* = 0.022) (GCC *vs.* ATA: OR 1.47, 95% CI 1.07 −2.01, *P* = 0.017) (Table 4). No other significant associations were observed between haplotype and Brucellosis risk.

**Table 3.** The general results of the association of IL-10 polymorphisms with Brucellosis risk

Comparison	Group	Test of association			Mode	Test of heterogeneity		
		OR	95% CI	*P*		*x*^2^	*P*	*I*^2^
−1082 (G →A)								
A *vs.* G	Overall	0.82	0.62–1.08	0.152	Fixed	6.94	0.139	42.4
	Caucasian	0.76	0.49–1.18	0.217	Random	4.90	0.086	59.1
	Asian	0.89	0.58–1.37	0.602	Fixed	1.78	0.182	44.0
AA *vs.* GG	Overall	1.51	0.87–2.60	0.142	Fixed	5.07	0.281	21.1
	Caucasian	1.36	0.58–3.18	0.484	Random	4.20	0.123	52.4
	Asian	1.95	0.83–4.58	0.124	Fixed	0.37	0.544	0
AA *vs.* GG/GA	Overall	0.80	0.49–1.30	0.360	Random	9.76	0.045	59
	Caucasian	0.62	0.39–0.98	0.043	Fixed	2.75	0.252	27.4
	Asian	1.42	0.39–5.23	0.598	Random	4.66	0.031	78.5
AA/GA *vs.* GG	Overall	0.72	0.39–1.32	0.291	Fixed	7.06	0.133	43.4
	Caucasian	0.96	0.44–2.09	0.916	Random	2.75	0.126	51.7
	Asian	**0.42**	**0**.**19–0**.**94**	**0.035**	Fixed	4.66	0.678	0
−819 (C → T)								
T *vs.* C	Overall	0.90	0.62–1.31	0.587	Random	11.71	0.02	65.8
	Caucasian	1.22	0.91–1.63	0.190	Fixed	1.28	0.528	0
	Asian	**0.60**	**0**.**44–0**.**82**	**0.001**	Fixed	0.02	0.889	0
TT *vs.* CC	Overall	0.71	0.28–1.81	0.471	Random	9.96	0.041	59.8
	Caucasian	1.22	0.56–2.65	0.615	Fixed	2.54	0.280	21.4
	Asian	**0.24**	**0**.**09–0**.**62**	**0.003**	Fixed	0.15	0.694	0
TT *vs.* CC/TC	Overall	0.68	0.28–1.65	0.393	Random	9.77	0.044	59.1
	Caucasian	1.15	0.60–2.23	0.670	Fixed	1.96	0.375	0
	Asian	**0.22**	**0**.**05–0**.**91**	**0.036**	Fixed	1.30	0.254	23.2
TT/TC *vs.* CC	Overall	1.33	0.73–2.44	0.353	Random	15.88	0.003	74.8
	Caucasian	1.31	0.90–1.90	0.160	Fixed	0.44	0.802	0
	Asian	1.62	0.19–13.80	0.658	Random	13.99	0	92.9
−592 (C → A)								
A *vs.* C	Overall	0.90	0.64–1.26	1.524	Fixed	7.56	0.109	47.1
	Caucasian	1.05	0.70–1.56	0.813	Fixed	2.21	0.331	9.6
	Asian	0.78	0.44–1.38	0.393	Random	3.55	0.059	71.9
AA *vs.* CC	Overall	0.58	0.32–1.06	0.076	Fixed	4.49	0.344	10.9
	Caucasian	0.77	0.32–1.85	0.564	Fixed	0.76	0.683	0
	Asian	0.48	0.14–1.67	0.248	Random	2.99	0.084	66.5
AA *vs.* CC/CA	Overall	0.63	0.37–1.07	0.086	Fixed	2.91	0.573	0
	Caucasian	0.86	0.38–1.94	0.710	Fixed	0.05	0.167	0
	Asian	0.51	0.19–1.33	0.168	Random	1.91	0.078	47.7
AA/CA *vs.* CC	Overall	0.80	0.51–1.26	0.345	Fixed	6.10	0.192	34.4
	Caucasian	0.78	0.34–1.80	0.567	Fixed	3.89	0.235	30.9
	Asian	0.80	0.40–1.67	0.535	Random	3.15	0.076	68.2

**Table 4.** The haplotype analysis of the association of IL-10 polymorphisms with Brucellosis susceptibility

Comparison	OR	95% CI	*P*
GCC/ACC	1.62	1.07–2.46	0.022
GCC/ATA	1.47	1.07–2.01	0.017
ACC/ATA	0.88	0.48–1.59	0.663

### Sensitivity analysis

Sensitivity analysis was executed for indicating single study's impact on the final result under every genetic model [16]. In the meta-analysis, whole studies could not affect the final results, manifesting the reliability and stability (figure not displayed).

### Publication bias

We could observe tiny asymmetrical funnel plots in Begg's funnel plot (*P* = 0.806) (figure not shown). Nevertheless, we could not observe apparent publication bias by Egger's test (*P* > 0.05).

## Discussion

Previous literatures have explored the connection of IL-10 polymorphism with Brucellosis susceptibility. In view of the inconsistent results and renewed information, we rigorously executed the present meta-analysis. For all we know, this research was firstly investigating the connection of IL-10 polymorphisms with Brucellosis risk. Our paper shows that IL-10-819 loci polymorphism is not relevant with susceptibility of Caucasian population but may contribute a decreased risk to Asian population. And neither IL-10-1082 loci nor 592 loci polymorphism is associated with Brucellosis risk. The present results show that IL-10-819 loci polymorphism may be connected with the difference of race. It is not difficult to understand that different ethnicity populations have different allele frequencies, especially in controls, reinforcing the necessity to perform subgroup analysis in the procedure of meta-analysis. Related to the present study, we made a conservative conclusion. Only two literatures were employed. Considering small quantity, a renewed meta-analysis should be urgently necessary after large and high-quality studies are reported.

The literatures which studied the connection of IL-10 polymorphisms with disease susceptibility were extensively reported. IL-10 polymorphisms were considered to be connected with multiple disease susceptibility such as ischemic stroke, pulmonary tuberculosis, HIV-1, nasopharyngeal carcinoma and multiple sclerosis, gastric cancer and inflammatory bowel disease [17–23]. The increased risk or reduced risk can be detected due to diverse reasons including studied races, sample size, genotyping methods and source of control population, which may lead to different conclusions.

Extensive changes have been established in cytokine frequency polymorphism in healthy population of different races, for instance, the -1082 loci polymorphism of IL-10. It has been investigated broadly. The prevalence rates of -1082 G allele vary among different countries and regions. The high prevalence rate of -1082 G allele can be found in Iranians and Norwegians, which can reach up to 42.5% and 48.9%, respectively and a low prevalence rate of -1082 G allele can be found in Japanese and Koreans, which is 3.8% and 13.0%, respectively [24–27]. So that it is necessary to take population-based studies into meta-analysis. In the present study, all the eligible studies were population-based, which reinforces the reliability of our results. According to the predefined evaluation standard, the eligible studies seemed to be ‘high-quality’ with scores ≥12. All of the controls were population-based from the same geographical area and matched age, sex and ethnicity with cases.

Some disadvantages should be mentioned and our conclusions should be interrupted with prudence. Primarily, the number of included studies was relatively small, which might bring about some bias and heterogeneity. Secondly, Brucellosis is a complex disease and its occurrence and development is affected by diversified elements.

In a word, this research makes clear that IL-10-819 loci polymorphism is not associated with Brucellosis risk of Caucasian population but may contribute a decreased risk to Asian population. And neither IL-10-1082 loci nor 592 loci polymorphism is associated with Brucellosis risk.

## Author contributions

Conceived and designed the experiments: YZ; Performed the experiments: XJ SY YZ; Analysed the data: SY YZ; Contributed reagents/materials/analysis tools: XJ SY YZ; Wrote the paper: XJ SY YZ.
